# Economic Burden of Stroke Disease: A Systematic Review

**DOI:** 10.3390/ijerph18147552

**Published:** 2021-07-15

**Authors:** Thinni Nurul Rochmah, Indana Tri Rahmawati, Maznah Dahlui, Wasis Budiarto, Nabilah Bilqis

**Affiliations:** 1 Department of Health Administration and Policy, Faculty of Public Health, Universitas Airlangga, Surabaya 60115, Indonesia; indana.tri.rahmawati-2018@fkm.unair.ac.id (I.T.R.); maznahd@ummc.edu.my (M.D.); wasisbudiarto@fkm.unair.ac.id (W.B.); nabilahbilqis@gmail.com (N.B.); 2 The Airlangga Centre for Health Policy Research Group, Surabaya 60115, Indonesia; 3 Centre of Population Health, Department of Social and Preventive Medicine, Faculty of Medicine, University of Malaya, Kuala Lumpur 50603, Malaysia

**Keywords:** economic burden of disease, length of stay, stroke, cerebrovascular accident

## Abstract

Globally, one of the main causes of non-communicable disease as a cause of death every year is stroke. The objective of this study was to analyze the burden in consequence of stroke. This research used a systematic review method. Furthermore, a search for articles was carried out in June–July 2020. Four databases were used to search articles from 2015 to 2020. Eligible studies were identified, analyzed, and reported following the Preferred Reporting Items for Systematic Reviews and Meta-Analysis (PRISMA) guidelines. The inclusion criteria were prospective cost studies, retrospective cost studies, database analysis, mathematical models, surveys, and COI studies that assess burden of stroke in primary and referral healthcare (hospital-based). The results showed that from four databases, 9270 articles were obtained, and 13 articles were qualified. A total of 9270 articles had the identified search keywords, but only 13 articles met the set criteria for inclusion. The criteria for inclusion were stroke patients, the economic burden of stroke disease based on cost of illness method, which is approximately equal to USD 1809.51–325,108.84 (direct costs 86.2%, and indirect costs 13.8%). Those that used the health expenditure method did not present the total cost; instead, only either direct or indirect cost of health expenditure were reported. For most hospital admissions due to stroke, LOS (length of stay) was the dominant cost. The high economic burden to manage stroke justifies the promotion and preventive efforts by the policymakers and motivates the practice of healthy lifestyles by the people.

## 1. Introduction

Cardiovascular disease (CVD) is a non-communicable disease and the world’s main cause of death (17.9 million deaths annually) [1]. Cardiovascular disease has been responsible for 37% of total mortality in Indonesia. Stroke is the leading cause of cardiovascular disease, followed by coronary heart disease and diabetes [2]. This shows that there is currently an epidemiological transition that has shifted the burden of disease from infectious diseases to non-communicable diseases [3].

Stroke is the leading cause of death and disability worldwide, and the economic costs of post-stroke care are enormous [4]. As of now, approximately 34% of the global total healthcare expenditure is spent on stroke. The average healthcare cost of stroke per person, including inpatient care, rehabilitation, and follow-up care, is estimated at USD 140,048 in the United States [5].

Stroke burden in people under the age of 65 has increased in recent decades. Currently, there is an alarming shift from the overall stroke burden to younger age groups, particularly in low-moderate-income countries. The worldwide incidence of stroke in the 20 to 64 years age group has increased by 25%, while the incidence of stroke is higher in men aged 55–75 years [6]. The epidemic increase in cardiovascular risk factors in young adults in regions such as Russia, China, and India has contributed to the increased stroke burden among the younger population [5]. The highest incidence rate of stroke occurred in Asia, a continent containing more than 60% of the world’s population; the second highest incidence rate of stroke was experienced by people in Eastern Europe, while the lowest was in central Latin America [6]. Mortality caused by stroke is higher in Asia than in Western Europe, America, or Australasia (similar to Eastern Europe).

In Indonesia, the national prevalence of stroke has increased from 0.7% in 2013 to 1.09% in 2018 [7]. The increase in the prevalence of cardiovascular disease results in the higher number of outpatient and inpatient services, as well as the economic impact that the state must manage through the National Health Insurance (JKN) Program. The data issued by the Social Security Agency for 2016 stated that stroke costs a service fee of USD 950,715, which makes non-communicable diseases a significant disease burden. Apart from medical expenses, people with cardiovascular disease will cause economic losses for the country’s productivity. Patients with cardiovascular disease generally have disabilities, making them unable to carry out their daily activities independently. This condition causes them to depend on other people to accompany them on their activities, including patients undergoing treatment [8].

Disease burden studies could help policymakers understand the economic costs of a particular disease. Such disease burden studies identify various cost components for a particular disease or disease-related complications in different sectors that might have been saved if the disease is not present. Moreover, disease burden studies have an essential role in public health to formulate and prioritize healthcare policies and allocate healthcare resources by estimating the total costs that can be incurred by the disease(s) [9].

Various indicators could describe the disease burden in the population. Epidemiological indicators include life expectancy, mortality rates, and the total of new and existing specific disease cases (e.g., incidence and prevalence) [10]. Epidemiological indicators show the value of the disability-adjusted life year (DALY) and quality-adjusted life year (QALY) from a disease. The use of inpatient and outpatient services is also an indicator of the disease burden [11] and economic loss measurements such as absences, incapacity to work, the use of medical facilities, and other related costs. The calculated economic components include direct medical costs, direct non-medical costs, and indirect costs outlined in the cost of illness (COI) and health expenditure (HE) methods.

This systematic review aimed to analyze the disease burden due to stroke. In this systematic review, some of the critical questions posed are: (1) analyzing the average length of hospitalization for stroke, and (2) the magnitude of economic losses due to stroke, including direct medical costs, direct non-medical costs, and indirect costs.

## 2. Methods

The method used in this systematic review consisted of a search strategy, inclusion and exclusion criteria, data extraction, and quality assessments of included studies.

### 2.1. Search Strategy

The literature search in this systematic review used databases with high- and medium-quality criteria, namely, Scopus, Science Direct, Proquest, and Springerlink journals. Search terms used burden of stroke disease studies were: “burden of disease”, “length of stay”, “cost of illness”, “burden of illness”, “cost of disease”, “cost of sickness”, “disease cost”, “economics burden of disease”, “sickness cost”, “cardiovascular disease”, “cerebrovascular accident (CVA)”, “cerebral accident”, “cerebrovascular apoplexy”, “cerebrovascular stroke”, “vascular accident”. The terms were matched with terms in the Medical Subject Heading (MeSH) database. The strategy used to search for articles was enacted by specifying the basis of economic evaluation studies considered in systematic reviews. These considerations included the participant or population, exposure, context (geographical health setting and culture), and outcome measures framework (PECOS) [12]. In addition, study selection was also reported in the Preferred Reporting Items for Systematic Reviews and Meta-Analysis (PRISMA) flowchart. The search strategy identified 9270 studies. The elimination of duplicates and title and abstract screening resulted in the removal of 6456 studies. The full-text screening was performed in 2814 studies that resulted in 2720 unrelated studies, which should be excluded. A total of 94 articles were assessed for eligibility and resulted in 81 articles that were excluded for being not related to stroke, its burden, and original articles. The search was carried out in June–July 2020, wherein the literature search was limited to the year of publication from 2015 to 2020. The searched articles for this systematic review had been confined to publications in the last 5 years (1 January 2015 to 22 March 2020) to capture the studies conducted in the era of multiple treatment options available which reduces the burden of recurrent stroke.

### 2.2. Inclusion and Exclusion Criteria

Inclusion and exclusion criteria were applied in economic evaluation to obtain relevant studies for further systematic review research. Inclusion and exclusion criteria were assessed according to PECOS. The inclusion criteria for participants or populations were studies that focused on stroke patients, while the exclusion criteria were absent. Furthermore, the inclusion criteria for exposure(s) were studies that examined stroke burden, while the exclusion criteria were absent. Furthermore, the inclusion criteria for context were studies covering primary and referral healthcare (hospital-based), and there were no exclusion criteria. In the outcome measures indicator, the inclusion criteria needed were studies that explained direct or indirect costs. Direct cost is expenditure related with expenditure for treatment and rehabilitation, as well as non-medical cost outside the health system such as patient transportation, the cost of informal care, or other expenses borne by the patient. Indirect costs are the loss of household productive labor time for patient and caregivers due to disability and mortality. This is mentioned in the Discussion section. There were no exclusion criteria. On the other hand, for study design and publication type, the inclusion criteria were all types of publications, including prospective cost studies, retrospective cost studies, database analysis, mathematical models, surveys, and COI studies. Meanwhile, the exclusion criteria were those that were not original articles. Regarding the publication years and language, those that had a publication year of 2015–2020 and were written in English were included, and there were no exclusion criteria.

### 2.3. Data Extraction and Data Analysis 

Data extraction was used to separate which data were involved in the research, consisting of several indicators used in assessing research articles. These indicators included the year of publication (2015–2020). The disease studied for this study was stroke. Research settings were grouped on the basis of the economic status of a country (lower-moderate income, upper-moderate income, and high income). Study designs that were included were prospective, retrospective, or cross-sectional studies. The calculation method used was cost of illness or health expenditure. The approach used was incidence-based or prevalence-based, a cost perspective on the healthcare system, third-party payers, participants, or society. This study was peer-reviewed by two people, namely, ITR and TNR. Keywords in this systematic review were adjusted to the Medical Subject Heading (MeSH). The keywords used to search were “length of stay”, “burden of disease”, “cost of illness”, “burden of illness”, “cost of disease”, “cost of sickness”, “disease cost”, “economics burden of disease”, “sickness cost”, “cardiovascular disease”, “cerebrovascular accident (CVA)”, “cerebral accident”, “cerebrovascular apoplexy”, “cerebrovascular stroke”, and “vascular accident”.

Data analysis was carried out by collecting and synthesizing information on general study characteristics (including country settings and economic status), methodological characteristics (study design, data sources, approaches, and calculated disease burden indicators), and estimated economic burdens (currency and year, cost components, cost perspective). Information was carried out by descriptively focusing on the burden of disease due to stroke at the household, health system, and community levels. Data were managed and analyzed using Microsoft Excel software.

### 2.4. Quality Assessments

Quality assessment has several eligibility criteria based on seven-question quality assessment tool adapted from Gheorghe’s study [13], in which it includes both the economic and epidemiological components. This instrument focuses on two aspects, namely, the design of economic and epidemiological studies (carried out in conjunction with economic studies). They include several questions that researchers use to assess the article content’s quality, appropriateness, and suitability. The economic aspects include: (1) explanation of data sources for expenditure, resource use, and unit costs; (2) transparency of data on costs and/or expenses; (3) calculation of productivity costs; (4) the results of the calculation of productivity costs; (5) analysis addresses uncertainty and/or heterogeneity, e.g., analysis on subgroups. In addition, the epidemiological aspects considered in this quality assessment had included: (1) sampling method to determine prevalence/incidence; (2) the source of prevalence data that contributes to the study’s internal validity. The total assessment of quality criteria categories performed were good (score 74–100%; fair (score 47–73%), and poor (20–46%).

## 3. Results

**Figure 1:**
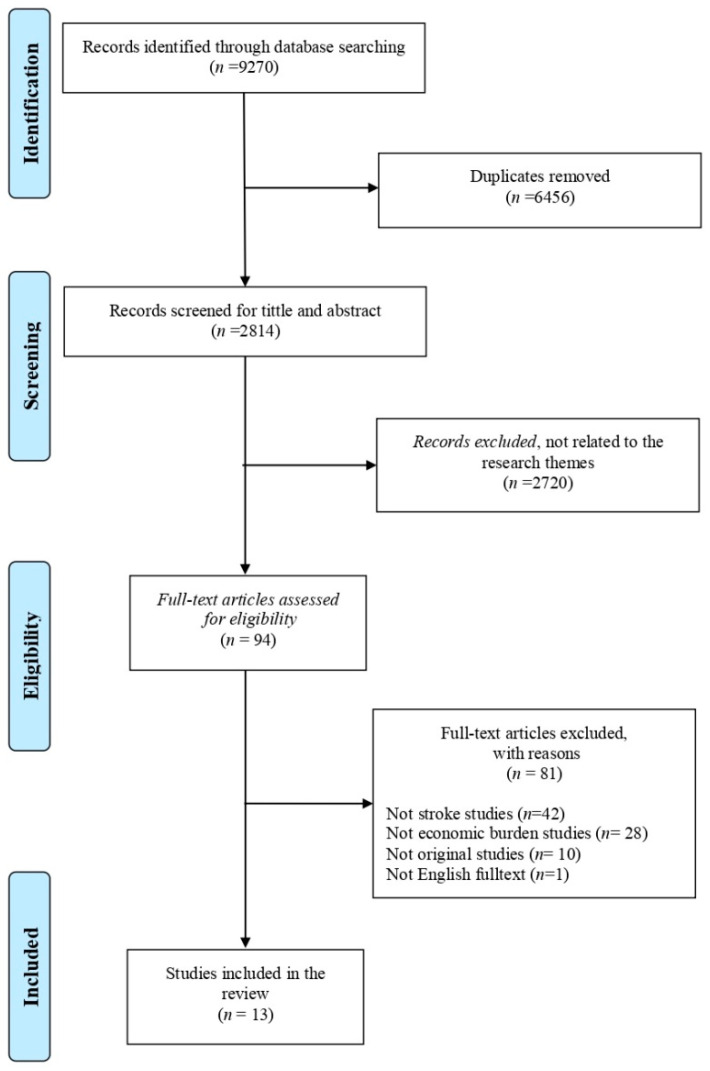
PRISMA flowchart.

Figure 1 presents a PRISMA flow chart that summarizes the inclusion and exclusion decisions made by the authors. Researchers found 9270 articles that matched the keywords, and search results obtained were then checked for duplications. At the initial identification stage, at least 6456 duplicate articles were deleted. A total of 2814 titles and abstracts were screened according to the theme (stroke burden). However, 2720 articles were excluded as they did not match the theme. On the basis of the title and abstract selection stages, we assessed 94 articles for their feasibility and conformity with the eligibility criteria. This stage resulted in 81 articles being excluded as they were not related to stroke (42 articles) nor disease burden (28 articles), were original articles (10 articles), and were not English full-texts (1 article). The application of these inclusion criteria resulted in 13 articles deemed suitable for systematic review research.

### 3.1. Quality of Included Studies

The quality of each article was determined using a seven-question quality assessment tool adapted from a previous study by Gheorghe et al. [14]. This instrument focused on two aspects, namely, epidemiological studies carried out in conjunction with economic studies. A summary of the results of this systematic review is presented (see Supplementary Materials Table S1). The majority of included studies did not meet all quality assessment criteria; however, none of the studies were classified as poor-quality studies. Five studies obtained a percentage of 100% good quality criteria (items 3, 4, 6, 8, and 11, see Supplementary Materials Table S2). One of the challenges associated with quality assessment was that quality was judged on the basis of published data only. There might be discrepancies between what has been reported and what has been done. The majority of the criteria that could not be met were the criteria regarding productivity costs due to the COI method, which did not calculate production costs, and thus many articles did not meet these criteria. In addition, for the epidemiological component, even though all articles met the criteria, there was no single article that presented the calculation of QALY or DALY.

### 3.2. Characteristics of Included Studies

The characteristics of included studies (*n* = 13) are shown in Table 1. The characteristics of the included studies are made to determine the study design, study scope, economic perspective, and the types of diseases required in the systematic review. Furthermore, from the included studies, 13 articles (100%) were studies on types of stroke [15,16,17,18,19,20,21,22,23,24,25,26,27]. Three types of study designs included a prospective cost study of five articles (38%) [15,22,23,24,26], a retrospective cost study of six articles (46%) [15,16,18,20,24,26], and a cross-sectional study of two articles (15%) [18,20]. The calculation method used was the COI in eight articles (62%) [15,17,19,20,23,25,26,27] and the HE method in five articles (38%) [16,18,21,22,24]. The approach taken was a prevalence-based approach in 11 articles (85%) [16,17,18,19,20,21,23,24,25,26,27] and 2 articles on the basis of incidence rate (15%) [15,22]. On the other hand, there were five articles (38%) with a societal economic perspective [18,20,21,22,25], and five articles (38%) with an economic perspective of healthcare system [15,19,23,25,27]. One article (8%) had a third-party payer perspective [16], and two articles (15%) had a participant and family perspective [17,24]. Meanwhile, countries used as research locations in terms of economic status were only in upper-middle-income and high-income countries whose articles were included in the criteria. In countries with upper-middle-income economic status, there was one article (8%) that fit the criteria in Lebanon, Colombia, China, Turkey, South Africa, and Brazil [15,16,19,23,26,27]. Furthermore, study locations in countries with high-income economic status and suitable articles were South Korea, Denmark, and the Netherlands, each with one article, respectively (8%) [17,20,25], while Sweden and United States had each published two articles (15%) [18,21,22,24].

Table 1 shows a summary of the disease burden indicators studied in each article, which included a total of 13 studies. The indicators studied in each article depended on the method used; either direct, indirect or both direct and indirect cost. On the basis of the results of these studies, one can group them according to the determined systematic review theme, namely, the burden of stroke. Presentation of results will be determined on the basis of length of hospitalization; calculation of economic losses in terms of methods, approaches, cost components, and economic perspectives; and the condition of economic loss due to stroke as per the included articles.

There are six countries with upper-middle-income economic status that were examined in terms of economic losses due to stroke, namely, Lebanon, Turkey, South Africa, Brazil, China, and Colombia, while the remaining countries have high-income economic status. Meanwhile, calculation using the COI or HE methods depends on the objective of the study. Both COI and HE may include either direct, indirect or both direct and indirect cost, therefore, a study can use a complex disease burden analysis using both the COI and HE methods [28].

### 3.3. Average Length of Stay for Stroke

Of the 13 selected articles, 3 articles examined the average length of hospitalization of stroke patients. The description of the length of stay for stroke patients can be seen in Table 2. 

Abdo [14] showed that in Lebanon, the average length of stroke at the hospital was 13–18 days. Another study conducted by İçağasıoğlu et al. (2017) [19] showed that in Turkey, the length of stay of stroke patients ranged from 0 to 75 days, with an average length of stay of 11–15 days. Meanwhile, a study conducted by Zhang et al. (2019) [27] showed that in China, stroke patients had an average of 27 days of hospitalization.

The mean LOS was higher in patients with intracerebral hemorrhage (ICH) compared to ischemic stroke (IS). Predictors of higher LOS were the National Institution of Health Stroke Scale (NIHSS), which had a high admission scale, patients at ICU, patients undergoing surgery, and patients with infectious complications [15].

On the other hand, İçağasıoğlu et al. (2017) [19] and Zhang et al. (2019) [27] did not describe in detail the causes of the length of hospitalization, and therefore more complete information could not be obtained by the researcher. Of the three articles, Turkey occupied the lowest position in the duration of hospitalization for stroke patients, while China occupied the highest position in the length of stroke hospitalization, which reached 27 days.

### 3.4. Cost Incurred by Stroke

There were 13 articles included to calculate the economic loss due to stroke. The articles came from different countries with different currencies and different years of research. In this systematic review, each result of economic loss calculation from the selected articles was adjusted to USD in 2020 (INT $2020). The results of the currency conversion for each cost are presented in Table 3.

Table 3 show the presentation of economic burden due to stroke was differentiated on the basis of country groups, namely, upper-middle-income and high-income countries. Each country group was also presented separately between the calculations on the basis of the COI method and the health expenditure method. The entire value of economic losses presented in this study was adjusted to USD in 2020. On the basis of 13 articles, there were 5 articles that analyzed the economic burden of stroke based on health expenditure, while the other 8 articles analyzed the economic burden of stroke on the basis of cost of illness. On the basis of the cost of illness method, we found that the largest cost component due to stroke was direct medical cost and indirect medical cost, accounting for 86.2% and 13.8% of the total cost, respectively. The economic burden of stroke disease in terms of cost of illness method was approximately equal to USD 1809.51–325,108.84. As for the articles that reported the cost on the basis of health expenditure method, only one article was found to have reported that the direct cost to treat stroke was, on average, USD 4905.33, while four articles measured the indirect cost, which was, on average, USD 2739.73.

The COI method is a method that is easy to implement but has the disadvantage of losing utility value due to disease [29]. On the other hand, the HE method can measure total health expenditure, representing the amount spent on healthcare and related activities (such as insurance administration) but lacks data that require more detailed and specific data on household spending, especially on indirect costs [24].

COI studies measure economic losses due to disease in certain populations [20]. COI studies generally involve two separate cost analyses, namely, direct costs and indirect costs. Direct costs are the value of the resources used for the treatment and rehabilitation of the person with the condition under study. Direct costs refer to all goods, services, and other resources consumed during the delivery of a health intervention for a particular disease. This includes money spent on hospital care, on the services of doctors and other medical professionals, medicines, equipment, and rehabilitation [19].

HE studies are used to calculate the costs incurred due to a disease. In this systematic review, we found that the seven articles that used the HE method focused on direct non-medical costs and indirect costs, which include informal caregiver costs and productivity lost. Informal caregivers are defined as care activities provided by relatives, with or without compensation. These activities include providing support for activities of daily living or instrumental activities of daily living [21]. Caregiver costs include personal travel time to visit sufferers and any expenses related to caregiving [18].

Loss of productivity is defined as the number of days of net sick leave and days of early retirement due to illness [30]. Meanwhile, the loss of productivity is assessed using the human capital approach with the assumption that production losses are assessed at market prices, namely, gross salary and payroll taxes. The cost of decreasing a person’s productivity is equal to the amount the employer is willing to pay for the production. This is assumed to be the average gross salary plus employer contributions [24].

## 4. Discussion

Stroke rapidly develops clinical signs of focal (or global) impairment of brain function, with symptoms lasting 24 h or more or leading to death without apparent cause other than a vascular cause [31]. To our knowledge, research is rarely conducted on the review of economic evaluation methods in various countries to see the calculation of economic losses using the COI and HE methods and to analyze the average length of stay for stroke patients.

The review identified several emerging patterns. We found that among published economic evaluations, there were no consistent outcome measures. The majority of the studies reported using the COI method, which varied on the basis of the point of view, data source, indirect cost criteria, and timeframe for cost calculation [32]. On the other hand, the use of the HE method was used to see the costs incurred by patients and families during healthcare, such as administrative costs and insurance in addition to the main variables analyzed such as the number of days at the hospital, home care services, the number of days with at least single contact with physicians, benefits of palliative home care, utilization of health services in the past weeks, and public healthcare expenditures [33]. This shows that the calculation of COI and HE could be carried out according to research needs, not with certainty. The heterogeneity of this outcome measure could prevent the comparison of the two methods to see the economic loss due to stroke in certain income groups, regions, or even countries.

On the basis of the results, the average LOS of stroke patients was 11 to 27 days. Several factors causing LOS were severity, stroke volume, infection, complications, demographic characteristics, and the presence of emergency or medical status of the patient. This is in line with research by Curtain et al. (2017) [34], who stated that stroke patients at Norwich University Hospital underwent an average hospitalization of 11 to 33 days. Furthermore, several factors affecting LOS for stroke patients included neurological lateralization, pre-stroke disability status, congestive heart failure, and age. Research conducted by Saxena and Prasad in 2016 also supported the results of this study [35], which stated that some causes of LOS for stroke patients were complications due to pressure sores and sepsis that needed them to stay for >7 days. Several studies suggested that a longer LOS leads to higher economic losses [36]. The less time the patient is hospitalized, the more effective and efficient services the hospital can provide. Meanwhile, inefficient services are one causing increased costs [37,38].

Direct costs incurred by the health system, society, families, and individual patients consisted of health and non-health costs. Health costs are defined as medical care expenditures for diagnosis, treatment, and rehabilitation. One of the challenges in calculating direct medical costs, particularly hospital costs, was that costs were often the only data available. Due to the nature of determining hospital costs, it often did not accurately reflect the underlying costs. Costs were often higher than unit costs [39], and the results of the systematic review showed that the direct medical costs due to stroke were USD 1593–34,138 [20,26]. In Indonesia, the average direct cost for stroke patients participating in the Social Security Agency at Dr. Moh. Saleh Hospital was USD 1895.36, with a minimum to the maximum value range of USD 463.43–5159.22 [40]. 

Meanwhile, for patients without insurance from the Social Security Agency, the total direct cost was USD 38,013.72. The systematic review results showed that the highest cost in direct medical costs was found in inpatient costs (almost 65%). This finding is in line with research by Ye et al. (2020) [41], who showed that inpatient care was the largest contributor to healthcare costs (70% of total costs), followed by outpatient services (11%) and skilled nursing care (8%).

Meanwhile, non-health costs are related to the consumption of non-healthcare resources such as transportation, household expenses, relocation, property loss, and informal care of any kinds. Estimation of the direct costs associated with chronic disease was higher than that related to an acute disease or infectious disease, for which methods of treatment and prevention were effective [42]. These non-medical direct costs were estimated to account for 50% or more of all direct costs [43]. This systematic review showed that the highest contributor to the high direct non-medical costs was the cost of caregivers (around 82%). This finding is in line with Alvarez-Sabín et al. [44], who showed that more than two-thirds of stroke care costs were spent for social costs, especially informal care.

Furthermore, indirect costs were part of the social welfare loss due to diseases. The remaining loss of welfare was represented by the loss of health time due to illness, suffering, and sadness [42]. For many diseases, the indirect costs were enormous and much greater than the direct medical costs [39]. The results of this systematic review discovered that the indirect costs of stroke were USD 957–15,823 [18,25]. In Indonesia, the average indirect cost of stroke patients participating in the Social Security Agency at Dr. Moh. Saleh Hospital was USD 223.08, with a minimum to the maximum value range of USD 102.18–486.33 [40].

Meanwhile, for patients without the insurance mechanism, the total indirect cost was USD 100,117.68. The current systematic review showed that the highest indirect costs were due to the cost of productivity lost spent for premature deaths (around 80%). This finding is in line with previous studies, which stated that 66% of the total costs of stroke in the United States was due to the indirect costs of premature deaths [45]. It is challenging to generalize the results of economic studies in various countries. Economic results were difficult to compare due to monetary issues (i.e., fluctuating exchange rates, the purchasing power of currencies), although purchasing power parity (PPP) can help compare results. It eliminates differences in price levels between countries, while regional demographic characteristics also influence resource consumption and unit costs. This resulted in the treatment costs varying widely between studies [46]. On the other hand, the epidemiological indicators found in the selected articles included prevalence and incidence. There were no articles that calculated DALY or QALY due to stroke in this systematic review. 

### 4.1. Strengths and Limitations

This study provided an overview of the economic costs due to stroke in several countries. This study provided an evidence base of disease economic losses in terms of each type of cost (direct medical costs, direct non-medical costs, and indirect costs). This study also described the differences in calculating the economic loss of disease on the basis of two methods, namely, COI and HE. In addition, this study also looked at the length of hospitalization for stroke patients. An economic perspective was also presented separately. This study was expected to be used as a consideration for policymaking, especially for priority allocation of health resources and preparation of disease prevention programs as in this study, the contributing component to cost for each disease was explained. However, the articles included in this study did not specifically discuss the economic costs of stroke in specific regions or groups of countries. This allowed for differences in the characteristics of each country, for example, the health system, health financing system, and sociodemographic characteristics of the people. This study also did not find indicators of DALY and QALY from the articles included. In writing this systematic review, the two reviewers (I.T.R. and T.N.R.) had different opinions, and thus they asked for the others reviewer’s opinion (M.D., W.B., and N.B., respectively). 

### 4.2. Implication 

Stroke is classified as a cardiovascular disease, a group of catastrophic diseases that require long treatment, and the cost is expensive. Nowadays, stroke attack many productive age groups, and therefore it can reduce productivity at work. Patients with disease generally have disabilities, being unable to carry out their daily activities independently. This makes them dependent on other people to be able to perform activities and undergo treatment. It can reduce the productivity of caregivers, who are usually not paid because they are members of their own family. Therefore, strengthening preventive efforts is important. The Ministry of Health has implemented several policies, including regular health checks, tobacco control, regular physical activity, healthy and balanced diet, adequate rest, and management of stress (Presidential Instruction Number 1 with the Healthy Living Community Movement, and Integrated Development Post for Non-Communicable Diseases (Ministry of Health of the Republic of Indonesia, 2017). In Indonesia, it is necessary to encourage the government to not only allocate attention and budget for BPJS Health, but also to pay attention and allocate a large budget for public health efforts as the spearhead of health promotion programs and primary prevention of disease in Indonesia. Reducing the burden of disease can be done with risk factor management as a preventive measure. 

A systematic review of the burden of disease due to stroke is needed, including two indicators, namely, epidemiological indicators (covering DALY and QALY) and economic indicators (covering direct and indirect costs). Further research is needed on the burden of CHD and stroke in Indonesia because they are still very rare.

## 5. Conclusions

This review showed that stroke is not only one of the major disease burdens but also that it incurs substantial economic loss to the country. The cost to manage stroke is high because of the long hospital care required, and even after discharge, continued health expenditures are necessary for its risk management and long rehabilitation. The high financial burden to manage stroke justifies the promotion and preventive efforts by the policymakers and motivates the practice of healthy lifestyles by the people. 

## 6. Patents

This research produced non-patent work as further research can find other differences in results.

## Figures and Tables

**Table 1:** A summary of the indicators of disease burden studied in selected articles (*n* = 13).

No.	Researcher and Year	Research Setting	Country Group	Approach				Source of Data	Indicator of Calculated Disease Burden
				Research Design	Calculation Method	Method	Cost Perspective		
1	Abdo, et al. (2018) [15]	Lebanon	Upper-middleincome	Prospective	Cost of illness (COI)	Incidence-based	Healthcare system	203 stroke patients	Direct medical cost
2	Camacho, et al. (2018) [16]	Colombia	Upper-middleincome	Retrospective	Health expenditure	Prevalence-based	Third-party payer	Data are provided by ACEMI, an association of Colombian private health insurance companies	Direct medical cost
3	Cha, Yu–Jin (2018) [17]	South Korea	High income	Retrospective	Cost of illness (COI)	Prevalence-based	Participant (patients) and families	Insurance claims data generated during 2015 in Korea (N = 515,848)	Direct medical cost, direct cost, indirect cost.
4	Ganapathy (2015) [18]	United States	High income	Cross-sectional	Health expenditure	Prevalence-based	Society	Internet survey data were collected from 153 caregivers of stroke patients	Indirect cost (productivity lost)
5	İçağasıoğlu, et al. (2017) [19]	Turkey	Upper-Middleincome	Retrospective	Cost of Illness (COI)	Prevalence-based	Healthcare system	84 stroke patients	Direct and Indirect cost
6	Jennum, et al. (2015) [20]	Denmark	High income	Cross-sectional	Cost of illness (COI)	Prevalence-based	Society	Records from the Danish National Patient Registry of 93,047 ischemic, 26,012 hemorrhagic, and 128,824 stroke patients were unspecified and compared with 364,433, 103,741, and 500,490 matched controls, respectively.	Direct medical cost
7	Joo, et al. (2017) [21]	United States	High income	Retrospective	Health expenditure	Prevalence-based	Society	Using the 2010 Health and Pension Study, data on un-institutionalized adults aged ≥ 65 years (n = 10,129) in 2015–2017	Indirect medical cost
8	Lekander, et al. (2017) [22]	Sweden	High income	Prospective	Health expenditure	Incidence-based	Society	47,807 patients were diagnosed with stroke during 2007–2010, allowing for two years of follow-up	Total cost
9	Maredza and Chola (2016) [23]	South Africa	Upper-middleincome	Prospective	Cost of illness (COI)	Prevalence-based	Healthcare system	A population of around 90,000 people living in the Agincourt sub-district of Mpumalanga province, northeast South Africa, covered by a demographic and health surveillance system (health and demographic surveillance system, HDSS)	Direct cost
10	Persson, et al. (2017) [24]	Sweden	High income	Prospective	Health expenditure	Prevalence-based	Participant (patients) and families	53 couples provided informal support, and 168 couples did not provide informal support	Indirect medical cost
11	Van Eeden, et al. (2015) [25]	The Netherlands	High income	Retrospective	Cost of illness (COI)	Prevalence-based	Society	395 stroke patients	Total cost
12	Vieira, et al. (2019) [26]	Brazil	Upper-middleincome	Prospective	Cost of illness (COI)	Prevalence-based	Healthcare system	173 stroke patients	Direct medical cost
13	Zhang, et al. (2019) [27]	China	Upper-middleincome	Retrospective	Cost of illness (COI)	Prevalence-based	Healthcare system	A total of 114,872 were hospitalized for five types of stroke	Direct medical cost

**Table 2:** Descriptions of length of hospitalization for stroke patients.

No.	Research Cited	Results of Research on Length of Hospitalization	Description on the Causes of Length of Hospitalization
1	Abdo et al. (2018) [15]	In Lebanon, the average stroke hospitalization was 13–18 days.	Predictors of higher LOS were high National Institution of Health Stroke Scale (NIHSS) at admission, ICU LOS, surgery, and infection complications.
2	İçağasıoğlu et al. (2017) [19]	In Turkey, the length of hospitalization of stroke patients ranged from 0 and 75 days, with a mean duration of 11–15 days.	NA
3	Zhang et al. (2019) [27]	In China, the average length of hospitalization in the hospital was 27 days.	NA

**Table 3:** Results of cost conversion due to stroke.

No.	Author	Country	Method	Calculated Indicator	Result							
					Economic Loss		Direct Medical Cost		Indirect Medical Cost		Indirect Cost	
					Research Result	USD in 2020	Research Result	USD in 2020	Research Result	USD in 2020	Research Result	USD 2020
1	Abdo, et al. (2018) [15]	Lebanon	Cost of Illness (COI)	Direct medical cost	N/A ^a^	N/A	USD 6961 (2016 INT $) ^b^	7536.43	N/A	N/A	N/A	N/A
2	Camacho et al. (2018) [16]	Colombia	Health expenditure	Direct medical cost	N/A	N/A	USD 4277–4846 (2012 INT $)	4905.33–4905.33	N/A	N/A	N/A	N/A
					N/A	N/A	USD 6245 (2012 INT $)	7162.45	N/A	N/A	N/A	N/A
3	Cha, Yu-Jin (2018) [17]	South Korea	Cost of illness (COI)	Direct medical cost, direct cost, indirect cost	USD 7247 (2015 INT $)	7931.80	N/A	N/A	N/A	N/A	N/A	N/A
4	Ganapathy (2015) [18]	United States	Health expenditure	Indirect cost (productivity lost)	N/A	N/A	N/A	N/A	N/A	N/A	Productivity loss of USD 269 for absenteeism and USD 598 for presenteeism. Total lost productivity of USD 835 per month (2012 INT $)	308.52 685.85 957.67
5	İçağasıoğlu et al. (2017) [19]	Turkey	Cost of illness (COI)	Direct and indirect cost	TL 17,253.50 (2014) ^c^	16,662.20	TL 8668 (2014)	8370.94	N/A	N/A	TL 10,800 (2014)	10,429.87
6	Jennum et al. (2015) [20]	Denmark	Cost of illness (COI)	Direct medical cost	EUR 10,772–13,888 (2009) ^d^	1701.07–2193.13	EUR 8297–10,088 (2009)	1310.23–1593.05	N/A	N/A	EUR 7377–10,720 (2009)	1164.94–1692.86
7	Joo et al. (2017) [21]	United States	Health expenditure	Indirect medical cost	N/A	N/A	N/A	N/A	N/A	N/A	USD 2883–5777 (2015 INT $)	3155.43–6322.90
8	Lekander et al. (2017) [22]	Sweden	Health expenditure	Total cost	EUR 10,000–120,000	5,367,715.39–64,412,584.69	N/A	N/A	N/A	N/A	N/A	N/A
9	Maredza and Chola (2016) [23]	South Africa	Cost of illness (COI)	Direct cost	N/A	N/A	USD 283,465 (2012 INT $)	325,108.84	N/A	N/A	N/A	N/A
10	Persson et al. (2017) [24]	Sweden	Health expenditure	Indirect Cost (Informal care cost)	N/A	N/A	N/A	N/A	N/A	N/A	EUR 991–25,127 (€ INT 2015)	123.80–3138.86
11	Van Eeden et al. (2015) [25]	The Netherlands	Cost of illness (COI)	Total cost	EUR 29,484 (2012)	25,043.49	EUR 18,068.2 (2012)	25,043.49	N/A	N/A	EUR 11,416 (2012)	15,823.18
12	Vieira et al. (2019) [26]	Brazil	Cost of illness (COI)	Direct medical cost	N/A	N/A	USD 2595–31532 (2016 INT $)	2809.51–34,138.58	N/A	N/A	N/A	N/A
13	Zhang et al. (2019) [27]	China	Cost of illness (COI)	Direct medical cost	N/A	N/A	USD 3212.1 (2013 INT $)	3620.45	N/A	N/A	N/A	N/A

^a^ N/A = not applicable; ^b^ USD = United States Dollar, INT = international, $ = dollar; ^c^ TL = Turkish lira; ^d^ € = Euro.

## Supplementary Materials

The following are available online at https://www.mdpi.com/article/10.3390/ijerph18147552/s1, Table S1: PRISMA Checklist [47], Table S2: Quality Assessment [14]. References [47,14] are cited in the Supplementary Materials.

## References

[1] World Health Organization (2018). Monitoring Health for The SDGs.

[2] The George Institute for Global Health (2017). Reducing the Burden of Cardiovascular Disease in Indonesia.

[3] Kementerian Kesehatan (2019). Laporan Tahunan Badan Penelitian Dan Pengembangan Kesehatan 2019.

[4] Johnson A.J., Dudley W.N., Wideman L., Schulz M. (2019). Physiological Risk Profiles and Allostatic Load: Using Latent Profile Analysis to Examine Socioeconomic Differences in Physiological Patterns of Risk. Eur. J. Environ. Public Health.

[5] Katan M., Luft A. (2018). Global Health Neurology. Semin. Neurol..

[6] Gorelick P.B. (2019). The global burden of stroke: Persistent and disabling. Lancet Neurol..

[7] Riskesdas K. (2018). Hasil Utama Riset Kesehata Dasar (RISKESDAS). J. Phys. A Math. Theor..

[8] Lilissuriani Saputra I., Ruby M. (2017). Perbedaan Biaya Riil Rumah Sakit dan Rarif INA-CBG untuk kasus katastropik dengan penyakit Jantung Koroner pada Pasien Rawat inap Peserta Jaminan Kesehatan Nasional di RSUZA. J. Kesehat Masy..

[9] Tremmel M., Gerdtham U.G., Nilsson P.M., Saha S. (2017). Economic burden of obesity: A systematic literature review. Int. J. Environ. Res. Public Health.

[10] Devleesschauwer B., Havelaar A.H., De Noordhout C.M., Haagsma J.A., Praet N., Dorny P., Speybroeck N. (2014). DALY calculation in practice: A stepwise approach. Int. J. Public Health.

[11] Zhu B., Wang Y., Ming J., Chen W., Zhang L. (2018). Disease burden of COPD in china: A systematic review. Int. J. COPD.

[12] The Joanna Briggs Institute (2014). The Systematic Review of Economic Evaluation Evidence.

[13] Katsanos A.H., Hart R.G. (2020). New Horizons in Pharmacologic Therapy for Secondary Stroke Prevention. JAMA Neurol..

[14] Gheorghe A., Griffiths U., Murphy A., Legido-Quigley H., Lamptey P., Perel P. (2018). The economic burden of cardiovascular disease and hypertension in low- and middle-income countries: A systematic review. BMC Public Health.

[15] Abdo R.R., Abboud H.M., Salameh P.G., Jomaa N.A., Rizk R.G., Hosseini H.H. (2018). Direct medical cost of hospitalization for acute stroke in lebanon: A prospective incidence-based multicenter cost-of-illness study. Inquiry.

[16] Camacho S., Maldonado N., Bustamante J., Llorente B., Cueto E., Cardona F., Arango C. (2018). How much for a broken heart? Costs of cardiovascular disease in Colombia using a person-based approach. PLoS ONE.

[17] Cha Y.J. (2018). The economic burden of stroke based on South Korea’s national health insurance claims database. Int. J. Health Policy Manag..

[18] Ganapathy V., Graham G.D., Dibonaventura M.D., Gillard P.J., Goren A., Zorowitz R.D. (2015). Caregiver burden, productivity loss, and indirect costs associated with caring for patients with poststroke spasticity. Clin. Interv. Aging.

[19] İçağasıoğlu A., Baklacıoğlu H.Ş., Mesci E., Yumuşakhuylu Y., Murat S., Mesci N. (2017). Economic burden of stroke. Turk. Fiz. Tip. Rehabil. Derg..

[20] Jennum P., Iversen H.K., Ibsen R., Kjellberg J. (2015). Cost of stroke: A controlled national study evaluating societal effects on patients and their partners. BMC Health Serv. Res..

[21] Joo H., Wang G., Yee S.L., Zhang P., Sleet D. (2017). Economic Burden of Informal Caregiving Associated With History of Stroke and Falls Among Older Adults in the U.S.. Am. J. Prev. Med..

[22] Lekander I., Willers C., Von Euler M., Lilja M., Sunnerhagen K.S., Pessah-Rasmussen H., Borgström F. (2017). Relationship between functional disability and costs one and two years post stroke. PLoS ONE.

[23] Maredza M., Chola L. (2016). Economic burden of stroke in a rural South African setting. eNeurol. Sci..

[24] Persson J., Levin L.Å., Holmegaard L., Redfors P., Svensson M., Jood K., Forsberg-Wärleby G. (2017). Long-term cost of spouses’ informal support for dependent midlife stroke survivors. Brain Behav..

[25] Van Eeden M., Van Heugten C., Van Mastrigt G.A.P.G., Van Mierlo M., Visser-Meily J.M.A., Evers S.M.A.A. (2015). The burden of stroke in the Netherlands: Estimating quality of life and costs for 1 year poststroke. BMJ Open.

[26] Vieira L.G.D.R., Safanelli J., Araujo T.D., Schuch H.A., Kuhlhoff M.H.R., Nagel V., Cabral N.L. (2019). The cost of stroke in private hospitals in Brazil: A one-year prospective study. Arq. Neuropsiquiatr..

[27] Zhang H., Yin Y., Zhang C., Zhang D. (2019). Costs of hospitalization for stroke from two urban health insurance claims data in Guangzhou City, southern China. BMC Health Serv. Res..

[28] Salvatore F.P., Spada A., Fortunato F., Vrontis D., Fiore M. (2021). Identification of health expenditures determinants: A model to manage the economic burden of cardiovascular disease. Int. J. Environ. Res. Public Health.

[29] Gochi T., Matsumoto K., Amin R., Kitazawa T., Seto K., Hasegawa T. (2018). Cost of illness of ishchemic heart disease in Japan: A time trend and future projections. Environ. Health Prev. Med..

[30] Banefelt J., Hallberg S., Fox K.M., Mesterton J., Paoli C.J., Johansson G., Gandra S.R. (2016). Work productivity loss and indirect costs associated with new cardiovascular events in high-risk patients with hyperlipidemia: Estimates from population-based register data in Sweden. Eur. J. Health Econ..

[31] Warlow C.P. (1998). Epidemiology of Stroke.

[32] Indrayathi P.A., Noviyanti R. (2016). Bahan Ajar Cost of Illness.

[33] Voda A.I., Bostan I. (2018). Public health care financing and the costs of cancer care: A cross-national analysis. Cancers.

[34] Curtain J.P., Yu M., Clark A.B., Gollop N.D., Bettencourt-Silva J.H., Metcalf A.K., Myint P.K. (2017). Determinants of length of stay following total anterior circulatory stroke. Geriatrics.

[35] Saxena R., Kishore P., Saxena S. (2017). To compare surgically induced astigmatism in SICS using two different incision sites–“superior vs. temporal”. Indian J. Clin. Exp. Ophthalmol..

[36] Hoogervorst-Schilp J., Langelaan M., Spreeuwenberg P., De Bruijne M.C., Wagner C. (2015). Excess length of stay and economic consequences of adverse events in Dutch hospital patients. BMC Health Serv. Res..

[37] Lubis I.K., Susilawati S. (2018). Analisis Length Of Stay (Los) Berdasarkan Faktor Prediktor Pada Pasien DM Tipe II di RS PKU Muhammadiyah Yogyakarta. J. Kesehat. Vokasional..

[38] Wartawan I.W. (2012). Analisis Lama Hari Rawat Pasien Yang Menjalani Pembedahan di Ruang Rawat Inap Bedah Kelas III RSUP Sanglah Denpasar Tahun 2011.

[39] Segel J.E. (2006). Cost-of-Illness Studies—A Primer; RTI-UNC Center of Excellence in Health Promotion Economics. https://pdfs.semanticscholar.org/3bbf/0a03079715556ad816a25ae9bf232b45f2e6.pdf.

[40] Munawwaroh A. (2019). Perhitungan Cost of Illness (COI) Pada Pasien Rawat Inap Penderita Stroke Peserta BPJS Di RSUD Dr. Mohamad Saleh Kota Probolinggo.

[41] Ye Z., Ritchey M., MacLeod K., Wang G. (2020). A Literature Review of the Direct Medical Costs of Stroke Across the Care Continuum in the US. Circ. Cardiovasc. Qual. Outcomes.

[42] Jo C. (2014). Cost-of-illness studies: Concepts, scopes, and methods. Clin. Mol. Hepatol..

[43] Ibrahim N., Pozo-Martin F., Gilbert C. (2015). Direct non-medical costs double the total direct costs to patients undergoing cataract surgery in Zamfara state, Northern Nigeria: A case series. BMC Health Serv. Res..

[44] Alvarez-Sabín J., Quintana M., Masjuan J., Oliva-Moreno J., Mar J., Gonzalez-Rojas N., Yebenes M. (2017). Economic impact of patients admitted to stroke units in Spain. Eur. J. Health Econ..

[45] Girotra T., Lekoubou A., Bishu K.G., Ovbiagele B. (2020). A contemporary and comprehensive analysis of the costs of stroke in the United States. J. Neurol. Sci..

[46] Belarmino A.D.C., Rodrigues M.E.N.G., Anjos S.D.J.S.B.D., Ferreira Júnior A.R. (2020). Collaborative practices from health care teams to face the covid-19 pandemic. Rev. Bras. Enferm..

[47] Shamseer L., Moher D., Clarke M., Ghersi D., Liberati A., Petticrew M., Shekelle P., Stewart L.A., PRISMA-P Group (2015). Preferred reporting items for systematic review and meta-analysis protocols (prisma-p) 2015: Elaboration and explanation. BMJ.

