# Supplementary material for: Economic Burden of Stroke Disease: A Systematic Review
Source: Int J Environ Res Public Health. 2021 Jul 15;18(14):7552. doi: 10.3390/ijerph18147552 (PMC8307880; doi:10.3390/ijerph18147552)
Supplement: Supplementary file 1 [file ijerph-18-07552-s001.zip › ijerph-1225166-supplementary.pdf]

Burden of Stroke Disease: A Systematic Review

Thinni Nurul Rochamh, Indana Tri Rahmawati, Maznah Dahlui, Wasis Budiarto and Nabilah Bilqis

Supplementary Materials

Table S1: PRISMA Checklist

Table S2: Quality Assessment

Table S1: PRISMA Checklist

Section and topic	Item No	Checklist item
**ADMINISTRATIVE INFORMATION**		
Title:		
Identification	1a	Identify the report as a protocol of a systematic review
Update	1b	If the protocol is for an update of a previous systematic review, identify as such
Registration	2	If registered, provide the name of the registry (such as PROSPERO) and registration number
Authors:		
Contact	3a	Provide name, institutional affiliation, e-mail address of all protocol authors; provide physical mailing address of corresponding author
Contributions	3b	Describe contributions of protocol authors and identify the guarantor of the review
Amendments	4	If the protocol represents an amendment of a previously completed or published protocol, identify as such and list changes; otherwise, state plan for documenting important protocol amendments
Support:		
Sources	5a	Indicate sources of financial or other support for the review
Sponsor	5b	Provide name for the review funder and/or sponsor
Role of sponsor or funder	5c	Describe roles of funder(s), sponsor(s), and/or institution(s), if any, in developing the protocol
**INTRODUCTION**		
Rationale	6	Describe the rationale for the review in the context of what is already known
Objectives	7	Provide an explicit statement of the question(s) the review will address with reference to participants, interventions, comparators, and outcomes (PICO)
**METHODS**		
Eligibility criteria	8	Specify the study characteristics (such as PICO, study design, setting, time frame) and report characteristics (such as years considered, language, publication status) to be used as criteria for eligibility for the review
Information sources	9	Describe all intended information sources (such as electronic databases, contact with study authors, trial registers or other grey literature sources) with planned dates of coverage
Search strategy	10	Present draft of search strategy to be used for at least one electronic database, including planned limits, such that it could be repeated
Study records:		
Data management	11a	Describe the mechanism(s) that will be used to manage records and data throughout the review
Selection process	11b	State the process that will be used for selecting studies (such as two independent reviewers) through each phase of the review (that is, screening, eligibility and inclusion in meta-analysis)
Data collection process	11c	Describe planned method of extracting data from reports (such as piloting forms, done independently, in duplicate), any processes for obtaining and confirming data from investigators
Data items	12	List and define all variables for which data will be sought (such as PICO items, funding sources), any pre-planned data assumptions and simplifications

Outcomes and prioritization	13	List and define all outcomes for which data will be sought, including prioritization of main and additional outcomes, with rationale
Risk of bias in individual studies	14	Describe anticipated methods for assessing risk of bias of individual studies, including whether this will be done at the outcome or study level, or both; state how this information will be used in data synthesis
Data synthesis	15a	Describe criteria under which study data will be quantitatively synthesised
	15b	If data are appropriate for quantitative synthesis, describe planned summary measures, methods of handling data and methods of combining data from studies, including any planned exploration of consistency (such as I^2, Kendall's τ)
	15c	Describe any proposed additional analyses (such as sensitivity or subgroup analyses, meta-regression)
	15d	If quantitative synthesis is not appropriate, describe the type of summary planned
Meta-bias(es)	16	Specify any planned assessment of meta-bias(es) (such as publication bias across studies, selective reporting within studies)
Confidence in cumulative evidence	17	Describe how the strength of the body of evidence will be assessed (such as GRADE)

Note: It is strongly recommended that this checklist be read in conjunction with the PRISMA-P Explanation and Elaboration (cite when available) for important clarification on the items. Amendments to a review protocol should be tracked and dated. **The copyright for PRISMA-P (including checklist) is held by the PRISMA-P Group and is distributed under a Creative Commons Attribution Licence 4.0.**

[47] Shamseer L, Moher D, Clarke M, Ghersi D, Liberati A, Petticrew M, Shekelle P, Stewart L, PRISMA-P Group. Preferred reporting items for systematic review and meta-analysis protocols (PRISMA-P) 2015: elaboration and explanation. BMJ. 2015 Jan 2;349(jan02 1):g7647.

Table S2: Quality Assessment

Quality Assessment Tool

Economic component [Yes, No, or Unclear]

Q1. Were data sources for expenditure, resource use and unit costs clearly explained?

Q2. Were cost and/or expenditure data transparently presented?

Q3. Have productivity costs been included?

Q4. If productivity costs were included, were results presented with and without productivity cost?

Q5. Did the analysis address uncertainty and/or heterogeneity? E.g. through sensitivity analysis or subgroup analysis

Epidemiologic component (if applicable) [Yes, No, or Unclear]

Q1. Is the patient sampling method appropriate for deriving nationwide estimates of incidence or prevalence?

Q2. Does the source of incidence or prevalence data contribute to the study's internal validity?

Reference:

[14] Gheorghe, A., Griffiths, U., Legido-Quigley, H., Murphy, A., Perel, P., & Lamptey, P. 2018. The economic burden of non-communicable diseases (NCDs) in low- and middle-income countries (LMICs): a systematic review. *BMC Public Health.* 18:975. doi: https://doi.org/10.1186/s12889-018-5806-x\

Summary Quality Assessment of Included Studies

No.	Criteria	Study Number												
		1	2	3	4	5	6	7	8	9	10	11	12	13
1.	Were data sources for expenditure, resource use and unit costs clearly explained?	YES	YES	YES	YES	YES	YES	NO	YES	YES	NO	YES	YES	YES
2.	Were cost and/or expenditure data transparentlpresented?	YES	YES	YES	YES	YES	YES	YES	YES	YES	YES	YES	YES	YES
3.	Have productivity costs been included?	NO	NO	YES	YES	NO	YES	YES	YES	NO	YES	YES	NO	NO
4.	If productivity costs were included, were results presented with and without productivity costs?	NO	NO	YES	YES	NO	YES	YES	YES	NO	YES	YES	NO	NO
5.	Did the analysis address uncertainty? E.g.through sensitivity analysis or subgroup analysis	YES	YES	YES	YES	NO	YES	YES	YES	YES	YES	YES	NO	YES
6.	Is the patient sampling method appropriate for deriving nationwide estimates of incidence / prevalence?	YES	YES	YES	YES	YES	YES	YES	YES	YES	YES	YES	YES	YES
7.	Does the source of incidence / prevalence data contribute to the study's internal validity?	YES	YES	YES	YES	YES	YES	YES	YES	YES	YES	YES	YES	YES

**Criteria Presentation**	71%	71%	100%	100%	57%	100%	86%	100%	71%	86%	100%	57%	71%
**Category**	M	G	G	G	M	G	G	G	M	G	G	M	M

Notes: M = Moderate; G = Good; Category: Moderate (Score 47% - 73%); Good (Score 74% - 100%)
